# Low Omega-3 Levels in the Diet Disturbs Intestinal Barrier and Transporting Functions of Atlantic Salmon Freshwater and Seawater Smolts

**DOI:** 10.3389/fphys.2022.883621

**Published:** 2022-04-28

**Authors:** Kristina Sundell, Gerd Marit Berge, Bente Ruyter, Henrik Sundh

**Affiliations:** ^1^ Swedish Mariculture Research Center, Department of Biological and Environmental Sciences, University of Gothenburg, Gothenburg, Sweden; ^2^ Norwegian Institute of Food, Fisheries and Aquaculture Research (Nofima), Nofima, Sjølsengen, Sunndalsøra, Norway; ^3^ Nofima, Ås, Norway

**Keywords:** omega-3, Atlantic salmon, intestinal transport, intestinal barrier function, tight junctions

## Abstract

Due to a limited access to marine raw materials from capture fisheries, Atlantic salmon feeds are currently based on mainly plant ingredients (75%) while only 25% come from traditional marine ingredients including marine fish meal and fish oil. Thus, current feeds contain less of the essential omega-3 fatty acids. The aim of the study was to assess the impact of different omega-3 levels in fish feed on intestinal barrier and transporting functions of Atlantic salmon freshwater and seawater smolts. Atlantic salmon were fed three levels of omega-3 (2, 1 and 0.5%) and fish performance was followed through smoltification and the subsequent seawater acclimation. Intestinal barrier and transporting functions were assessed using Ussing chamber methodology and combined with transcript analysis of tight junction related proteins and ion transporters. A linear decrease in growth was observed with decreasing omega-3 levels. Low (0.5%) inclusion of omega-3 impaired the barrier function of the proximal intestine compared to 2% inclusion. Further, low levels of omega-3 decrease the transepithelial electrical potential across the epithelium indicating disturbed ion transport. It can be concluded that low dietary levels of omega-3 impair somatic growth and intestinal function of Atlantic salmon.

## Introduction

The long chain omega-3 fatty acids EPA (20:5 n-3; eicosapentaenoic acid) and DHA (22:6 n-3; docosahexaenoic acid) are essential for the health and normal development of Atlantic salmon (Ruyter et al., 2000a, 2000b; Bou et al., 2017a, 2017b). These fatty acids play a key role in maintenance of proper structure and function of the cell membranes, being parts of the amphipathic lipids constituting the membranes and precursors for signaling molecules like eicosanoids and resolvins as well as regulators of the transcription of genes involved in lipid metabolism (Miyazaki and Ntambi, 2008; Smith and Murphy, 2015). Fish oil and fish meal have traditionally been the main sources of EPA and DHA for the Atlantic salmon (*Salmo salar* L.) aquaculture industry (Ytrestøyl et al., 2015; Aas et al., 2019). Due to a limited access to marine raw materials as well as ethical considerations regarding capture fisheries and the utilization of human food grade fish for fish feed production, salmon feed have developed to substitute marine ingredients with plant-based alternatives and currently most salmon feeds contain ca. 25% marine ingredients and 75% plant ingredients (Aas et al., 2019).

The intestine is a multifunctional organ, where the intestinal epithelium absorbs ions, fluid and nutrients in a controlled manner while efficiently restricts harmful agents in the intestinal lumen to reach the circulatory system (Veillette et al., 1993, 1995; Sundell et al., 2003; Tipsmark et al., 2010a, 2010b; Sundell and Rønnestad, 2011; Sundell and Sundh, 2012; Sundh et al., 2014; Sundh and Sundell, 2015). The Atlantic salmon intestine thus act as a selective primary barrier towards the environment. The intrinsic intestinal barrier consists of the epithelial cells (enterocytes), connected at the apical side through intercellular protein complexes collectively known as the tight junction (TJ). The TJ consists of a range of different integral membrane proteins (e.g. occludin and claudins) and associated cytoplasmic proteins such as ZO-1 (Günzel and Fromm, 2012). TJs provide two types of barrier functions, 1) a fence, that sustain epithelial polarity by restricting lateral diffusion of membrane proteins from the apical to the basolateral membrane and vice versa and 2) a gate, that maintain a high selectivity for passive transfer of substances (diffusion) through the paracellular pathway (Günzel and Fromm, 2012). The gate function is mainly determined by the type of claudin isoforms present in the TJ (Anderson and Van Itallie, 2009; Günzel and Yu, 2013). In fish, 63 claudin isoforms have been described in 16 different species (Kolosov et al., 2013). Recent findings suggest that at least 26 claudin isoforms are expressed in tissues of the Atlantic salmon (Tipsmark et al., 2008), where claudin-15, and 25b are suggested to be specific for the intestine (Tipsmark et al., 2010a).

Intestinal barrier and transporting functions are essential for fish health and welfare (Segner et al., 2012; Sundh and Sundell, 2015). Impaired intestinal barrier function, *i.e.* a leaky gut, is correlated to intestinal inflammation and a disturbed intestinal immune barrier of Atlantic salmon (Knudsen et al., 2008; Sundh et al., 2010, 2019; Niklasson et al., 2011) and may result in decreased disease resistance (Sundh et al., 2009). In this perspective, the composition of the feed is essential for the intestinal health as the intestine is the first organ to be exposed to the digested feed. A suboptimal feed with poor nutritional value or which contain anti nutrient factors impairs the intestinal barrier function (Knudsen et al., 2008; Vidakovic et al., 2015; Estensoro et al., 2016).

To ensure a healthy salmon, there has been an increasing focus on studying the effects of the current development of commercial salmon feed with lower and lower levels of EPA and DHA. In trout deprived of essential fatty acids, reduced apical uptake of Na^+^ (Nonnotte et al., 1987) and modulated osmoregulatory ability after transfer to seawater have been reported (Finstad and Thomassen, 1991). Further, a lack of essential fatty acids is reported to reduce growth and increase lipid accumulation in the intestine (Olsen et al., 1999, 2003; Bou et al., 2017a) as well as reduce the stress coping ability of salmonids (Bou et al., 2017b; Løvmo et al., 2021). Low dietary levels of EPA and DHA also lead to reduced levels of EPA and DHA in the enterocyte plasma membrane phospholipids (PLs), whereas the pro-inflammatory n-6 fatty acids levels increase (Bou et al., 2017a; 2017b). In gilthead seabream, intestinal barrier functions are negatively affected by low levels of omega-3 in feed (Estensoro et al., 2016). If and how the intestinal health of the Atlantic salmon is affected by low levels of EPA and DHA in the feed is currently unknown.

The aim of the present study was to assess the impact of different omega-3 levels in fish feed on general fish health and welfare and intestinal barrier and transporting functions of Atlantic salmon fresh- and seawater smolts. Fish growth and mortality as well as plasma ion levels are well known indicators for fish health and welfare and osmoregulatory capacity (Noble et al., 2018). Further, as the intestine is the first organ to potentially be affected by the fatty acid composition of the feed, the fatty acid composition of the phospholipids (PLs) in intestinal tissue was determined. Intestinal barrier and transporting functions were assessed using the Ussing-chamber methodology. Finally, the expression of key TJ proteins was determined using qPCR.

## Materials and Methods

### Experimental Fish and Rearing Conditions

Atlantic salmon parr with an average weight of 60 g, originating from the SalmoBreed strain, were used for the trial. At the beginning of the trial, 120 fish were stocked in each of nine experimental units, 500 L cylindrical fiberglass tanks supplied with recirculating freshwater at 12.4 (std 0.6)°C. The fish were initially held at short day-length (12 h light:12 h dark) for 6 weeks, followed by another 4 weeks with 24 h light to induce smoltification. When fish were ready for transfer to seawater (salinity 32–33 ppt and temperature 13.0 (std.1.0)°C), all fish groups were moved to nine larger, 3,2 m^3^ tanks for the seawater period of the trial. The entire feeding trial was done in the Nofima Centre for Recirculation in Aquaculture (NCRA) in Sunndalsøra, Norway, using recirculated water. The RAS systems are described in detail by (Terjesen et al., 2013). The experiment was terminated 96 days after seawater transfer. The experiment was performed in compliance with the Norwegian national regulation for use of experimental animals (NARA, FOR-2015-06-18-761) and the National Guidelines for Animal Care and Welfare published by the Norwegian Ministry of Education and Research and approved by Norwegian Food Safety Authority (FOTS); approval nr. 11666.

### Experimental Diets and Feeding

Three experimental diets with different levels of the omega-3 fatty acids EPA and DHA were produced at Nofimas Feed Technology Center in Bergen, Norway. Composition of the diets is presented in Table 1, and fatty acid profiles in Table 2.

**TABLE 1:** Composition of experimental diets.

	2% EPA + DHA	1% EPA + DHA	0.5% EPA + DHA
g 100 g^−1^			
Fish meal^a^	16.00	16.00	16.00
Wheat^b^	7.10	7.10	7.10
Wheat gluten^c^	14.40	14.40	14.40
Soy protein concentrate^d^	19.00	19.00	19.00
Corn gluten^e^	10.00	10.00	10.00
Horse beans^f^	4.00	4.00	4.00
Oil mix^g^	20.40	20.40	20.40
Soy lecithin^h^	1.00	1.00	1.00
Vitamin mix^i^	2.00	2.00	2.00
Mono sodium phosphate^j^	2.50	2.50	2.50
Carophyll pink. 10% Ax^k^	0.05	0.05	0.05
Yttrium oxide^l^	0.01	0.01	0.01
l-lysine^m^	1.40	1.40	1.40
Mineral mix without zink^n^	0.555	0.555	0.555
Zink^o^	0.087	0.087	0.087
Threonine^p^	0.20	0.20	0.20
Betafine^q^	0.50	0.50	0.50
dl-methionine^r^	0.80	0.80	0.80
Chemical content, g 100 g^−1^			
Dry matter	94.4	94.3	94.0
Total lipid	26.7	26.8	26.8
Crude protein	45.5	45.3	45.1
Ash	6.9	6.9	6.9
Energy, MJ kg^−1^	23.0	23.0	23.0

a Fish meal—LT, Fishmeal, Vedde, Norge.

b Wheat, Møllerens, Norge.

c Wheat gluten—Vital wheat gluten, Tereos Syral, Belgium.

d SPC – Soy protein concentrate, Agrokorn, Denmark.

e Corn gluten—Corn gluten meal, Agrokorn, Denmark.

f Horse beans, Socomac Rouen, France.

g Rapeseed oil, Emmelev, Denmark, and Fish oil, Pelagia, Norway.

h Soy lecithin—Denothin 62, Denofa, Norway.

i Vitamin-mix—Vilomix, Norway.

j MSP – Vilomix, Norway.

k Carophyll pink, 10% Ax—DSM, France.

l Yttrium oxide—VWR, Norway.

m l-lysine—Vilomix, Norway.

n Mineralmix uten Zn—Vilomix, Norway.

o Organic Zn—Alltech, Norway.

p Threonine—Vilomix, Norway.

q Betafine—Vilomix, Norway.

r dl-methionine—Vilomix, Norway.

**TABLE 2:** Fatty acid content in experimental diets, g ∗ 100 g^−1^.

	2% EPA + DHA	1% EPA + DHA	0.5% EPA + DHA
C 14:0	0.66	0.30	0.14
C 16:0	1.81	1.48	1.35
C 18:0	0.31	0.34	0.36
C 20:0	0.06	0.08	0.09
C 22:0	0,04	0,05	0,05
C 24:0	0.03	0.02	0.02
Sum SFA^a^	2.96	2.32	2.04
C 16:1 n-7	0.50	0.26	0.14
C 18:1 n-9	4.95	7.40	8.54
C 18:1 n-7	0.41	0.51	0.54
C 20:1 n-9	1.18	0.66	0.40
C 22:1 n-7	0.06	0.03	0.01
C 22:1 n-11	1.59	0.73	0.30
C 24:1 n-9	0.00	0.02	0.02
Sum MUFA^b^	8.82	9.67	9.99
C 18:2 n-6	2.71	3.65	4.23
C 18:3 n-3	0.85	1.24	1.40
C 20:4 n-3	0.08	0.04	0.02
C 20:2 n-6	0.02	0.02	0.02
C 20:4 n-6	0.03	0.02	0.01
C 20:5 n-3	0.90	0.48	0.26
C 22:5 n-3	0.09	0.06	0.04
C 22:6 n-3	0.86	0.45	0.24
Sum PUFA^c^	5.57	5.96	6.24
Sum EPA + DHA	1.76	0.92	0.50
Sum n-3	2.79	2.26	1.97
Sum n-6	2.77	3.69	4.26
n6/n3 ratio	0.99	1.63	2.16

a Sum SFA includes C15:0, C17:0.

b Sum MUFA includes C14:1n-5, C16:1n-9, C16:1n-5, C17:1n-7, C20:1n-11.

c Sum PUFA includes C16:3n-4.

Each of the test diets were fed to triplicate groups of fish, through both periods of the experiment. Feeding was planned according to estimated growth rate, but adjusted according to observed appetite. The fish were fed approximately 15–20% in excess to secure *ad libitum* conditions. Fish were weighed at start, at transfer to seawater, and at termination of the seawater feeding period. Specific growth rate (SGR) was calculated according to the following formula:

SGR, % pr day = (lnW_2_-lnW_1_) (t)^−1^*100; where W_1_ and W_2_ are average body weight (g) at start and end of the trial, and t is the number of days in the trial. Mortality was registered daily in each tank, dead fish were removed and individually weighed. Total accumulated mortality was calculated in % of initial number of fish.

### Sampling

Fish were randomly and gently netted and instantly euthanized by an overdose of MS-222. Samples were taken for analysis of plasma ions, fatty acid composition of phospholipids, intestinal gene expression and Ussing chamber analysis of intestinal functions, as described below.

### Plasma Ions

Whole blood was sampled from the caudal vessels using Vacuette^®^ heparinized vacuum tubes (Greiner Bio-One, Kremsmuster, Austria) from five fish in each tank in freshwater 2–4 days before seawater transfer and at 5 and 68 days after transfer to seawater. The blood was centrifuged at 3500 g for 10 min, plasma separated and stored at −80°C until further analyses. Photometric analyses of Na^+^, Cl^−^ and K^+^ were done by Nofima, Sunndalsøra (Pentra C400 HORIBA; HORIBA medical, Montpellier, France).

### Fatty Acid Composition of Phospholipids

Samples of proximal intestines (the region from just after the pyloric caeca to the thicker distal intestine with complex circular folds) for lipid analyses were collected from five fish per tank, after 5 and 68 days in seawater. A pooled sample of five fish per tank was homogenized making in total three pooled samples per dietary group (*n* = 3). Total lipids were extracted by the Folch method (Folch et al., 1957). The chloroform phase with the total lipid fraction was used to analyse the phospholipids (PLs). The chloroform was evaporated under nitrogen gas and then the total lipid fraction was re-dissolved in hexane. PLs were thereafter separated from non-polar lipids using thin layer chromatography with petroleum ether: diethyl ether: acetic acid (113:20:2, by vol.) as mobile phase. The PL fraction was visualised by spraying the TLC plates with 0.2% (w/v) 2′,7′-dichlorofluorescein in methanol, and by comparing the retention factor (Rf) with known standards under UV light. The PL fraction was thereafter scraped off and used for analysis of fatty acid composition. Briefly, the extract was dried under nitrogen gas and methyl esters of fatty acids were made with 2′,2′- methoxypropane, methanolic HCl and benzene by following the method of Mason and Waller (Mason and Waller, 1964). The fatty acid methyl esters were separated in a gas chromatograph (Hewlett Packard 6,890) equipped with a split injector by using an SGE BPX70 capillary column (length, 60 m; internal diameter, 0.25 mm; and film thickness, 0.25 μm; SGE Analytical Science), flame ionisation detector and HP Chem Station software. The carrier gas was helium, and the temperature of injector and detector was 280°C. The oven temperature was increased from 50 to 180°C at the rate of 10°C/min, and then increased to 240°C at a rate of 0.7°C/min. Standards were used to identify individual fatty acid methyl esters. The relative amount of each fatty acid was expressed as a percentage of the total amount of fatty acids in the analysed sample, and the absolute amount of fatty acids per g of tissue was calculated using the fatty acid 23:0 methyl ester as the internal standard.

### Intestinal Function

Assessment of intestinal function using the Ussing chamber technique were performed on four fish from each tank (*n* = 12) at peak smolt in freshwater, 2–4 days before transfer to seawater, and 94–96 days in seawater. The body cavity was opened laterally and the intestine, from just posterior to the last pyloric caeca to the rectum was carefully removed. The intestine was separated into a proximal region with simple folds only and a distal part with simple and complex folds, and placed in an ice-cold salmon Ringer solution (140 mM NaCl, 2.5 mM KCl, 15 mM NaHCO_3_, 1.5 mM CaCl_2_, 1 mM KH_2_PO_4_, 0.8 mM MgSO_4_, 5 mM HEPES, 10 mM d-glucose, 0.5 mM l-lysine and 20 mM l-glutamine (pH 7.8) for freshwater smolts, and 150 mM NaCl, 2.5 mM KCl, 2.5 mM CaCl_2_, 1.0 mM MgCl_2_, 7.0 mM NaHCO_3_, 0.7 mM NaH_2_PO_4_, 5 mM HEPES, 10 mM d-glucose, 0.5 mM l-methionine and 20 mM l-glutamine (pH 7.8) for the smolts in seawater aerated with 99.7% air and 0.3% CO_2_.

The intestinal segments were mounted into modified Ussing chambers, filled with the appropriate salmon Ringer solution and the temperature was kept at the acclimation temperature of the fish by a cooling mantle. Mixing and oxygenation was maintained by a gas lift, driven by a gas mixture of 99.7% air and 0.3% CO_2_. The exposed tissue surface area was 0.75 cm^2^ and the half-chamber volume 4 ml. After mounting, the intestinal segments were allowed 60 min of recovery for stabilisation of the electrical parameters. To assess the paracellular permeability of the intestinal segments the transepithelial electrical resistance (TER) as well as the apparent permeability (P_app_) of the hydrophilic marker molecule ^14^C-mannitol were monitored using an Ussing chamber system as previously described (Sundell and Sundh, 2012). Together with TER, continuous monitoring of the transepithelial electrical resistance (TEP; mV) and short-circuit current (SCC; µA/cm^2^) was used as monitoring of preparation viability and indicators of net ion distribution and net ion flow respectively. After the 60 min acclimation period the experiment was started by renewing the Ringer solution on the serosal side, and replacing the Ringer solution on the mucosal side with Ringer containing 40 kBq ml^−1^ of the hydrophilic marker molecule ^14^C-mannitol (0.1 mCi ml^−1^ and 55.5 mCi mmol^−1^; PerkinElmer, Boston, MA, United States). For the freshwater smolts also 0.14 MBq mol^−1^ of ^3^H-l-lysine in presence of 0.5 mM unlabelled l-lysine was added to the freshwater Ringer. l-lysine could not be used in seawater acclimated fish due to supply issues. The fish sampled in seawater had the same activity as well as unlabelled amino acid concentrations added to the seawater Ringer, but using l-methionine instead. A 100 µL sub-sample was taken from the mucosal half chamber at t = 0 and from the serosal Ringer at time points 0, 20, 25 30, 60, 80, 85 and 90 min. The serosal samples were replaced with equal volume of fresh Ringer solution to maintain same hydrostatic pressure across the intestinal tissue. Radioactivity was assessed in a liquid scintillation counter (Wallac 1409, Turku, Finland) after adding 5 ml Ultima Gold™ (PerkinElmer, Boston,United States). Apparent permeability coefficient for mannitol (P_app;_ cm/s) was calculated using Eq. 1

Papp = dQ/dT  × 1/ACo  (1)

where d*Q*/d*T* is the appearance rate of the molecule in the serosal compartment of the Ussing chamber, A is the area (cm^2^) of intestinal surface exposed in the chamber and C_o_ is the initial concentration (mol ml^−1^) on the mucosal side. Amino acid transport was calculated using Eq. 2

(dQ/dT)/A  (2)

where d*Q*/d*T* is the appearance rate of the nutrient in the serosal compartment of the Ussing chamber (mol/min) and A is the area (cm^2^) of intestinal surface exposed. The electrical parameters TER, TEP and SCC are reported as mean value of the last five measurements between 130–150 min.

### qPCR

From fish sampled for Ussing chamber analysis, a small ring of proximal and distal intestine respectively (FW: *n* = 6; SW: *n* = 12) were cut open, cleaned from feces and the mucosa was scraped off using objective glass (100–200 mg) and placed into tubes that contained 1 ml of RNAlater^®^ (Sigma-Aldrich Co.), which was stored at −80°C after 24 h in +4°C. Upon mRNA extraction, mucosa samples were separated from RNAlater^®^ and lysed in 600 μL RLT Plus buffer with 5 mm steel beads using a TissueLyser II homo-geniser (Qiagen NV, Hilden, Germany) for two cycles of 3 min at 25 rotations sec^−1^. Samples were centrifuged for 3 min at 17,000 g and supernatant was pipetted into spin columns for mRNA extraction using RNeasy^©^ Plus Mini kits (Qiagen NV), according to the manufacture’s manual. RNAse-free water was used to elute the mRNA and quantity was determined by Nanodrop (Thermo Fisher Scientific Inc.). RNA quality was assessed on random subsamples using a 2,100 Bioanalyzer system (Agilent Technologies). Samples were diluted twice to obtain 1,000 ng of mRNA. The cDNA was synthesized by reverse transcriptase using iScript™ Synthesis kits (Bio-Rad Laboratories Inc., Copenhagen, Denmark) with random primers in 20 μL reactions in one cycle of 5 min at 25°C, 30 s at 42°C and 5 min at 85°C in a thermocycler (Bio-Rad Lab Inc.). The primers sequences used for *claudin-15* and *-25b* was previously published by (Tipsmark et al., 2010a), *tricellulin* and *occludin* in (Tipsmark and Madsen, 2012) and *nka α1c* by (Nilsen et al., 2007). Primers were verified using NCBIs Primer-BLAST tool (https://www.ncbi.nlm.nih.gov/tools/primer-blast) and obtained from Eurofins MWG operon (Ebersberg, Germany). Efficiencies of each primer pair were confirmed to be between 90 and 105% using a dilution series of 2–50 ng cDNA pooled from six random samples. For each sample, duplicate 10 μL reactions of 10 ng cDNA, 0.5 μM of each primer pair (0.3 μM for β-actin) and SYBRGreen Supermix (Bio-Rad Lab Inc.) were pipetted into a reaction plate. Plates were analysed for 40 cycles with initial denaturation at 95°C for 3 min, followed by 40 cycles of 95°C for 10 s and 57–61°C for 30 s using a CFX Connect Real-time PCR Detection System (Bio-Rad Lab Inc.). The reference gene, ribosomal protein L 23 (*rpl23*), was used (Stefansson et al., 2012) and did not differ between freshwater or seawater acclimated fish [threshold cycle (CT) value 20.2 ± 0.18 and 20.2 ± 0.14 respectively (mean ± SEM)] in this study. The ratio of relative expression between the target and reference gene was calculated based on the CT: relative ex-pression = 2-[C (target)−CTT (reference)] using the 2−ΔC T′ method (Livak and Schmittgen, 2001).

### Statistical Analyses

Data on specific growth rate were first analysed by a One-way ANOVA, to assess the effect of diet. Secondly, a linear regression model was applied, using level of EPA + DHA in the diet as the independent variable. Individual data on plasma ion levels were analysed by a mixed model with diet as fixed effect and tank as random effect (GLM procedure in SAS 9.4 Software, SAS Institute Inc., Cary, NC, United States). The levels of fatty acids in intestinal phospholipids were analysed by a One-way ANOVA. Electrical parameters and mannitol data from the Ussing chamber analyses and transcript levels were analyzed using a 2-way ANOVA including omega-3 levels and salinity as fixed factors and analysing possible interactions between factors. Significant main effects were analysed using Tukey´s multiple comparisons test and significant interactions using Sidak´s multiple comparisons test. Amino acid transport was analysed in freshwater and seawater separately using 2-way ANOVA with intestinal region and omega-3 levels as fixed factors. A non-parametric frequency analysis was performed on the distribution of serosa positive/negative TEP (Chi-square test for trends). Normality and homoscedasticity were analysed using Shapiro-Wilk test and Spearmans´s test respectively. Data not fulfilling the assumptions for statistical analyses were log-transformed. All analyses were performed using GraphPad Prism version 8.0.2 for Windows, GraphPad Software, San Diego, California United States, www.graphpad.com. Significant effects were considered at *p* < 0.05.

## Results

### Growth, Mortality and Plasma Ions

The mortality during the entire experiment was 2.9%, and there were no differences between dietary treatments. The statistical analyses by One-way ANOVA did not reveal any significant differences in specific growth rate (SGR) between the three dietary groups (*p* = 0.13). However, the linear regression analysis revealed that there was a significant (*p* = 0.03) linear increase in SGR in relation to increased dietary content of EPA and DHA. SGR increased from 1.08 in the lowest (0.5%) to 1.11 in the intermediate (1%) and further to 1.19 in the high (2%) omega-3 diet. Levels of plasma ions did not show any dietary effects either in freshwater or the two samplings in seawater (Table 3).

**TABLE 3:** Plasma ion levels (mM) of Atlantic salmon fed different levels of omega-3, in freshwater smolts 2–4 days before seawater transfer, 5 and 68 days after transfer of fish to seawater.

	2% EPA + DHA	1% EPA + DHA	0.5% EPA + DHA	*p*-value
Freshwater smolt				
Cl^−^	101.8 ± 1.7	101.3 ± 1.6	105.3 ± 1.6	0.15
Na^+^	157.7 ± 0.7	158.0 ± 0.7	159.4 ± 1.6	0.38
K^+^	3.8 ± 0.2	3.1 ± 0.3	3.9 ± 0.5	0.06
5 days in seawater				
Cl^−^	137.9 ± 0.8	139.4 ± 0.8	138.7 ± 0.6	0.81
Na^+^	157.8 ± 0.6	157.9 ± 0.4	157.9 ± 0.7	0.99
K^+^	3.3 ± 0.2	3.1 ± 0.1	3.2 ± 0.4	0.95
68 days in seawater				
Cl^−^	131.4 ± 0.6	134.3 ± 1.0	131.7 ± 0.5	0.12
Na^+^	161.0 ± 0.8	161.4 ± 0.8	160.4 ± 0.6	0.79
K^+^	1.5 ± 0.1	1.6 ± 0.2	1.5 ± 0.2	0.91

### Fatty Acids in Intestinal Phospholipids

In samples collected 5 days after transfer to seawater, there were no change in the relative distribution between total saturated (SAT), monounsaturated (MUFA) and polyunsaturated (PUFA) fatty acids between the dietary groups (Table 4). However, the levels of EPA and 18:1n-9 (oleic acid; OA) in percentage of total fatty acids was higher and the level of 20:4n-6 (arachidonic acid; ARA) was lower, in fish from the high omega-3 diet group as compared to the two other dietary groups. At the end of the trial (Table 5), there were still no change in the relative distribution of total SAT between the dietary groups. But also at this time point the relative levels of EPA and DHA increased and the relative levels of the fatty acids 18:1n-9, 18:2n-6 (alpha-linolenic acid; ALA) and ARA as well as the n-6/n-3 ratio decreased, with increasing dietary levels of EPA and DHA (regression analyses).

**TABLE 4:** Fatty acid (FA) composition (% of total) in the phospholipid (PL) fraction of Atlantic salmon proximal intestine first sampling (5 days after transfer to seawater).

	FA in Proximal intestine			Aanova	Regression omega-3 in feed	
	2% EPA + DHA	1% EPA + DHA	0.5% EPA + DHA	*p*-value	*p*-value	R-square
∑ SFA	30.2 ± 0.34	30.2 ± 0.37	29.3 ± 0.46	0.24	0.23	0.19
18:1 n-9	11.5 ± 0.24^b^	13.7 ± 0.22^a^	14.6 ± 0.67^a^	0.006	0.0008	0.82
∑ MUFA	19.4 ± 0.36	20.3 ± 0.21	21.2 ± 1.05	0.23	0.08	0.37
18:2 n-6	4.0 ± 0.20	4.3 ± 0.36	4.4 ± 0.34	0.66	0.35	0.12
20:4 n-6	2.7 ± 0.02^c^	3.4 ± 0.07^b^	4.5 ± 0.05^a^	<0.0001	<0.0001	0.90
20:5 n-3	4.4 ± 0.17^a^	3.6 ± 0.05^b^	3.6 ± 0.15^b^	0.006	0.003	0.75
22:6 n-3	32.5 ± 0.38	31.5 ± 0.36	29.7 ± 1.30	0.12	0.05	0.45
∑ PUFA	51.7 ± 0.23	51.9 ± 0.22	52.7 ± 0.63	0.27	0.17	0.25
∑ EPA DHA	37.0 ± 0.44^a^	35.1 ± 0.32^ab^	33.3 ± 1.15^b^	0.04	0.008	0.66
n6/n3	0.2 ± 0.01^b^	0.2 ± 0.01^ab^	0.3 ± 0.02^a^	0.01	0.003	0.73

Data are shown as mean values using tank as a statistical unit (*n* = 3, being each sample represented by a pool of five fish) with their standard errors. Different superscript letters indicate statistically significant differences with one-way anova (*p* < 0.05). The table also shows the *p*-value and R-square for the regression analyses of increasing omega-3 fatty acids in feed.

**TABLE 5:** Fatty acid (FA) composition (% of total) in phospholipid (PL) fraction of Atlantic salmon proximal intestine second sampling (68 days after transfer to seawater.

	FA in Proximal intestine			Anova	Regression omega-3 in feed	
	2% EPA + DHA	1% EPA + DHA	0.5% EPA + DHA	*p*-value	*p*-value	R-square
∑ SFA	28.5 ± 0.61	28.6 ± 1.78	26.4 ± 0.86	0.42	0.32	0.14
18:1 n-9	13.5 ± 0.17^c^	17.3 ± 0.38^b^	18.8 ± 0.40^a^	<0.0001	<0.0001	0.95
∑ MUFA	18.6 ± 0.40^b^	21.8 ± 0.39^a^	23.2 ± 0.57^a^	0.001	0.0001	0.90
18:2 n-6	6.8 ± 0.16^b^	8.9 ± 0.43^a^	9.8 ± 0.16^a^	0.0007	<0.0001	0.91
20:4 n-6	1.7 ± 0.38	2.6 ± 0.50	3.2 ± 0.22	0.08	0.02	0.57
20:5 n-3	9.0 ± 0.82^a^	6.0 ± 0.34^b^	5.4 ± 0.15^b^	0.005	0.01	0.80
22:6 n-3	23.8 ± 0.26^a^	18.5 ± 1.57^b^	18.2 ± 1.04^b^	0.02	0.006	0.68
∑ PUFA	48.1 ± 0.13	45.4 ± 2.38	47.3 ± 1.78	0.54	0.60	0.04
∑ EPA DHA	32.9 ± 0.89^a^	24.5 ± 1.83^b^	23.5 ± 1.19^b^	0.005	0.002	0.78
n6/n3	0.3 ± 0.02^b^	0.5 ± 0.03^a^	0.6 ± 0.02^a^	0.0002	<0.0001	0.92

Data are shown as mean values using tank as a statistical unit (*n* = 3, being each sample represented by a pool of five fish) with their standard errors. Different superscript letters indicate statistically significant differences with one-way anova (*p* < 0.05). The table also shows the *p*-value and R-square for the regression analyses of increasing omega-3 fatty acids in feed.

### Intestinal Barrier Function

**FIGURE 1:**
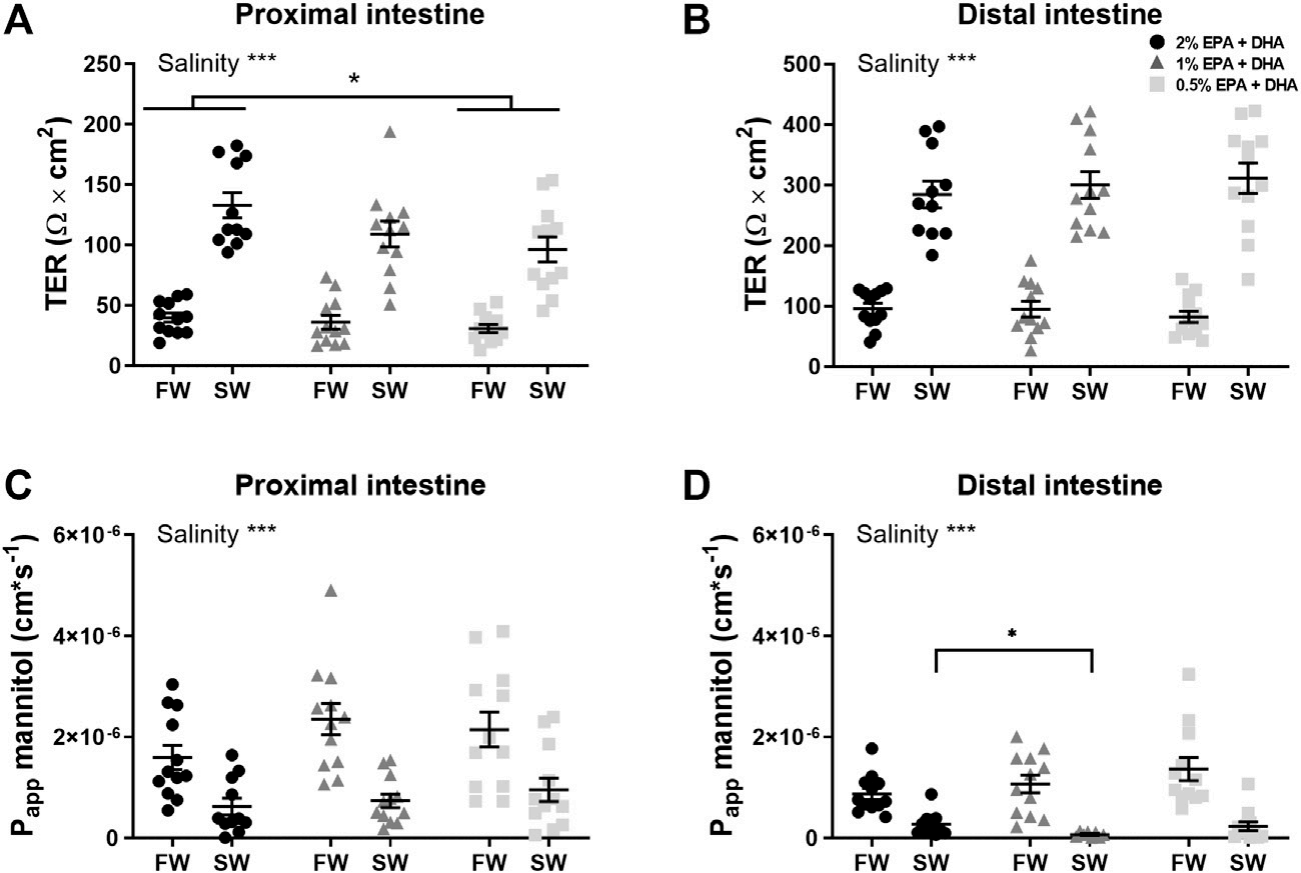
Barrier function assessed as transepithelial electrical resistance (TER; **(A,B)** and apparent permeability coefficient (P_app_) for ^14^C-mannitol **(C,D)** in proximal and distal intestine of freshwater (FW) smolts and seawater (SW) acclimated Atlantic salmon fed different levels of omega-3. Statistics: 2-way ANOVA where main effects were compared with Tukey´s multiple comparisons test. Significant effects from the post-hoc test are reported as *p < 0.05 and ***p < 0.001. Data are presented as individual values with the horizontal line representing the mean and the vertical lines SEM.

A functional intestinal barrier is essential for fish health and welfare (Sundh and Sundell, 2015). Intestinal barrier function towards ions and small water-soluble substances were assessed as transepithelial electrical resistance (TER; Ω*cm^2^) and the apparent permeability coefficient (P_app;_ cm/s) for ^14^C-mannitol, respectively. Main effects were observed for TER in the proximal intestine (Figure 1A) for diet (*p* < 0.05) and salinity (*p* < 0.001) while no interaction was observed. Tukeys’ multiple comparisons test revealed significantly lower TER in the 0.5% group compared to 2% group (*p* < 0.01), while the 1% omega-3 was similar to the other diets groups (*p* = 0.1022). In the distal intestine (Figure 1B), no effects were observed regarding diet (*p* = 0.908) whereas the TER was significantly increased by salinity (*p* < 0.001) in all dietary groups as there were no interactions (*p* = 0.539). The diffusion rate of ^14^C-mannitol (P_app_) in the proximal intestine (Figure 1C), was not affected by omega-3 levels, but was significantly reduced after seawater acclimation (*p* < 0.001). In the distal intestine (Figure 1D), a significant interaction between diet and salinity was observed (*p* < 0.05) and the following post-hoc test showed that the P_app_ in the 1% omega-3 were lower compared to 2% omega-3. In addition, similarly as for the proximal intestine, the P_app_ in the distal region was significantly lower after seawater acclimation (*p* < 0.001).

### Intestinal Transport

**FIGURE 2:**
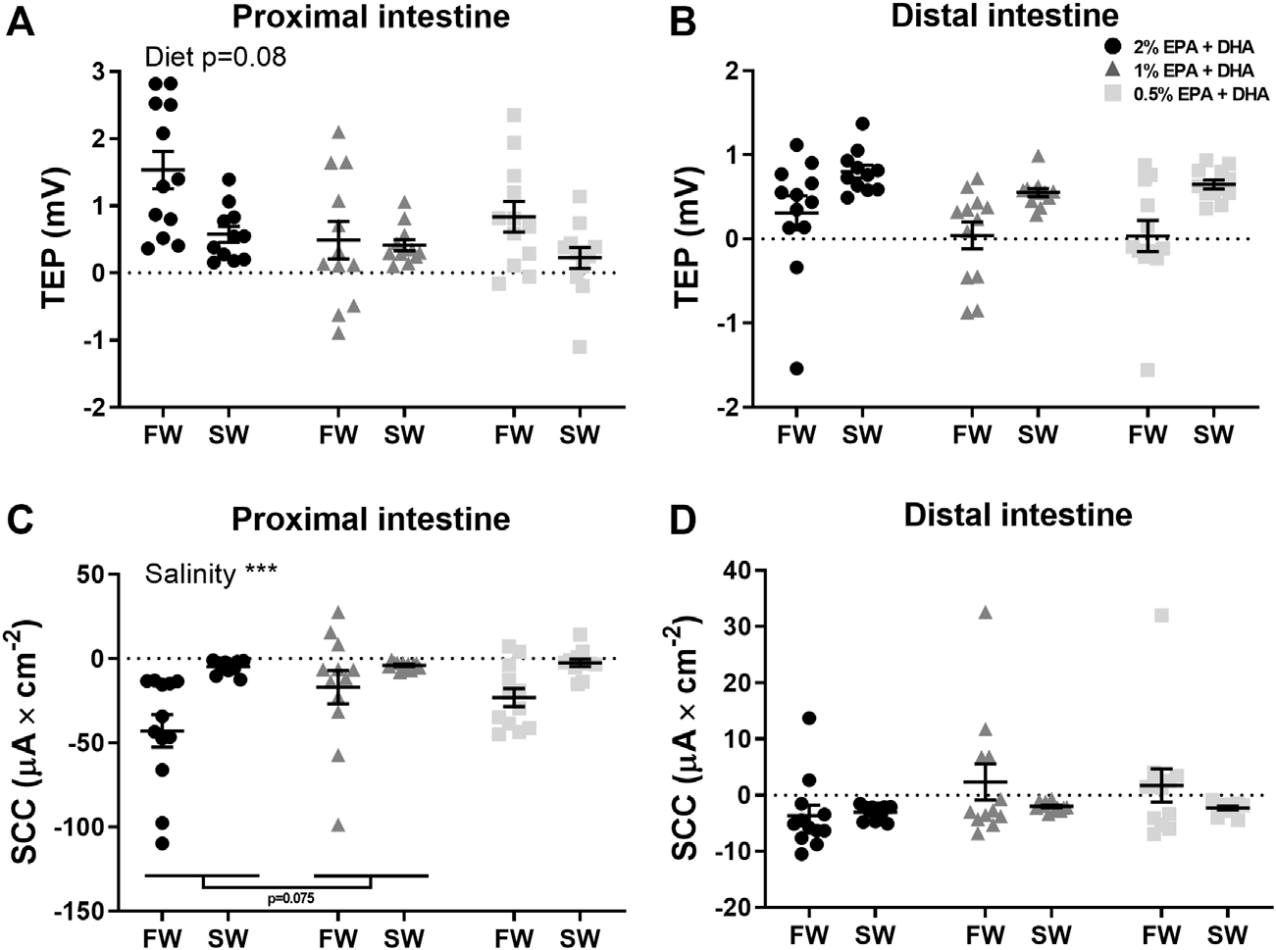
Active intestinal ion transport assessed as transepithelial potential difference (TEP; **(A,B)** and short-circuit current (SCC; **(C,D)** in freshwater (FW) smolts and seawater (SW) acclimated Atlantic salmon fed different levels of omega-3. Statistics: 2-way ANOVA where main effects were compared with Tukey´s multiple comparisons test. Significant effects from the post-hoc test are reported as *p < 0.05 and ***p < 0.001. Data are presented as individual values with the horizontal line representing the mean and the vertical lines SEM.

In the proximal intestine (Figure 2A), overall effects on TEP were observed for salinity (*p* < 0.001) while the effect of diet was not significant (*p* = 0.08). In the distal intestine (Figure 2B), the TEP was in general more serosa-positive in seawater compared to in freshwater. Further, a marked diet effect could be observed in the net-ion distribution across both intestinal regions, as decreased omega-3 levels reduced the occurrence of serosa-positive TEP’s in the distal intestine of freshwater fish and in the proximal intestine of seawater fish (*p* < 0.05; chi-square, Table 6). In neither the proximal (Figure 2C) nor the distal intestine (Figure 2D), any statistical differences were found in response to diets regarding SCC. Salinity significantly increased (less negative) SCC in the proximal intestine (*p* < 0.001), while no effects of salinity were observed in the distal intestine. Amino acid transport was assessed as an indicator for possible effects on active nutrient transport. In freshwater, the l-lysine transport was not affected by diet but was significantly higher in the proximal compared to the distal region (0.4 ± 0.02 nmol*min^−1^*cm^−2^ and 0.1 ± 0.005 nmol*min^−1^*cm^−2^ respectively; *p* < 0.001). Similarly, l-methionine transport in seawater was not affected by diet but was significantly higher in the proximal compared to the distal region (0.5 ± 0.07 nmol*min^−1^*cm^−2^ and 0.09 ± 0.009 nmol*min^−1^*cm^−2^ respectively; *p* < 0.001).

**TABLE 6:** Number of intestines displaying serosa positive TEP values in Atlantic salmon freshwater smolts and seawater post-smolts.

	Proximal intestine			Chi-square	Distal intestine			Chi-square
	2% EPA + DHA	1% EPA + DHA	0.5% EPA + DHA		2% EPA + DHA	1% EPA + DHA	0.5% EPA + DHA	
Freshwater	12 of 12	9 of 12	10 of 12	0.24	10 of 12	8 of 12	5 of 12	0.03
Seawater	12 of 12	12 of 12	9 of 12	0.03	12 of 12	12 of 12	12 of 12	1

### qPCR

**FIGURE 3:**
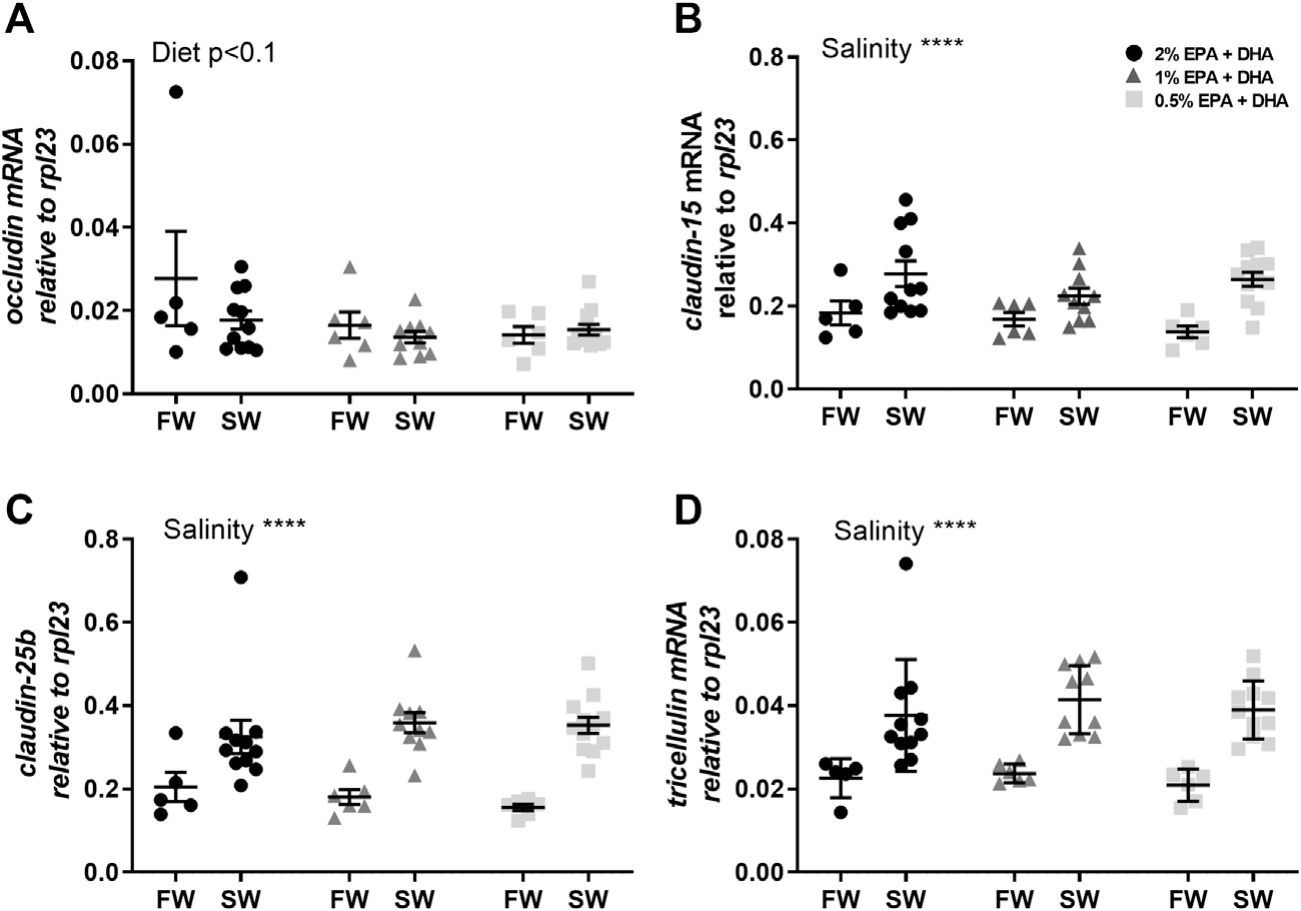
Transcript levels of **(A)** *occludin*, **(B)** *claudin-15*, **(C)** *claudin-25b* and **(D)** *tricellulin* in the proximal intestine of freshwater (FW) smolts and seawater (SW) acclimated Atlantic salmon fed different levels of omega-3. Statistics: two-way ANOVA. Significant effects are reported as *p < 0.05 and ***p < 0.001. Data are presented as individual values with the horizontal line representing the mean and the vertical lines SEM.

**FIGURE 4:**
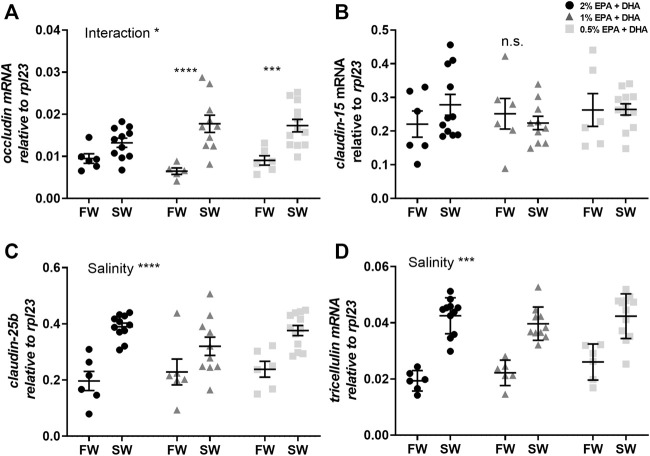
Transcript levels of **(A)** *occludin*, **(B)** *claudin-15*, **(C)** *claudin-25b* and **(D)** *tricellulin* in the distal intestine of freshwater (FW) smolts and seawater (SW) acclimated Atlantic salmon smolts fed different levels of omega-3. Statistics: two-way ANOVA where significant interaction was analysed using Sidak’s multiple comparisons test. Significant effects are reported as *p < 0.05 and ***p < 0.001. Data are presented as individual values with the horizontal line representing the mean and the vertical lines SEM.

In the proximal intestine, *occludin* transcript levels were not statistically affected by diet (*p* < 0.1) or salinity (Figure 3A). The other tight junction proteins, *claudin-15* (Figure 3B), *claudin-25b* (Figure 3C) and *tricellulin* (Figure 3D), were significantly upregulated after in seawater compared to freshwater smolts (*p* < 0.001). In the distal intestine, the expression of *occludin* was significantly upregulated by salinity in the 1 and 0.5% omega-3 groups, but not in the 2% omega-3 group (Figure 4A). The c*laudin-25b* (Figure 4C) and *tricellulin* (Figure 4D) were significantly upregulated in seawater compared to freshwater smolts (*p* < 0.001). No effects were observed for *claudin-15* (Figure 4B).

**FIGURE 5:**
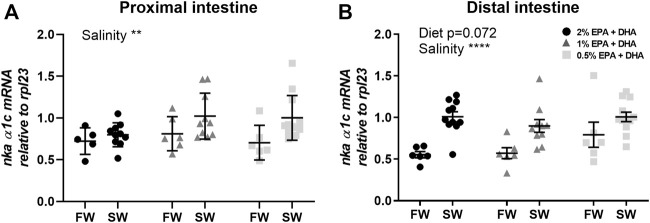
Transcript levels of Na^+^/K^+^-ATPase (*nka*) isoform *α1c* in the proximal **(A)** and distal **(B)** intestine of freshwater (FW) smolts and seawater (SW) acclimated Atlantic salmon fed different levels of omega-3. Statistics: 2-way ANOVA where main effects were compared with Tukey´s multiple comparisons test. Significant effects from the post-hoc test are reported as *p < 0.05 and ***p < 0.001. Data are presented as individual values with the horizontal line representing the mean and the vertical lines SEM.

The Na^+^/K^+^-ATPase (NKA) is the main driver of active ion, fluid and nutrient transport in the intestinal epithelium. The *nka α1c* transcript levels were upregulated after seawater acclimation in both proximal (Figure 5A) and distal intestine (*p* < 0.01; Figure 5B) while no significant diet effects were observed.

## Discussion

Total SGR in the current study was well in line with previously published studies on Atlantic salmon (Føre et al., 2016), and increasing dietary level of EPA and DHA from 0.5% up to 2% did cause a linear increase in SGR. Earlier studies have shown differing results regarding the effect of omega-3 fatty acids on growth rate dependent on environment and size of the fish (Ruyter et al., 2000a; Rosenlund et al., 2016; Bou et al., 2017a, 2017b). Ion levels were in the normal range of Atlantic salmon smolts in freshwater and both shortly (5 days) after transfer to seawater and at termination of the feeding trial. Thus, low omega-3 levels do not seem to lead to any sever osmoregulatory problems. However, decreased growth rate with decreasing omega-3 levels indicate that the physiology of the fish was affected, and may be a result of an increased allostatic load on the fish.

The significant increase in TER of both intestinal regions after seawater acclimation is in line with previous observations of salmonids acclimated to seawater (Sundell et al., 2003; Sundell and Sundh, 2012). The reduction in TER in both freshwater and seawater smolts in the low omega-3 diet group, on the other hand, clearly indicate that the intestinal barrier is impaired. The role of the observed marked increase in TER after seawater acclimation is not fully characterized but has been hypothesized to have a role in the intestine when the intestinal fluid uptake increases in seawater (Sundell and Sundh, 2012). According to this hypothesis, a high osmotic coupling compartment must be created in the lateral intercellular space (LIS) to attract fluid from the lumen across the intestinal epithelium to the blood side (Grosell, 2010; Sundell and Sundh, 2012; Whittamore, 2012). The high osmolality is mainly created by laterally located NKAs resulting in high Na^+^ levels in the LIS. The increased TER in SW aids in maintaining the high intercellular osmolality. The decrease in TER, as seen in the 0.5% omega group, most likely reflects an increased Na^+^ permeability across the TJ. A consequence therefore of the impaired barrier towards ions in the low dietary levels of EPA and DHA may be impaired intestinal fluid uptake.

The proximal region of the intestine, together with the pyloric caeca, is the most important regions for nutrient uptake while both proximal and distal intestinal regions are important for fluid uptake. The TEP in the current study were in general serosa positive, which is in agreement with previous studies on freshwater and seawater acclimated Atlantic salmon (Sundh et al., 2010). The seawater acclimated Atlantic salmon, on the other hand, often deviates from most stenohaline seawater teleosts, normally showing a serosa negative TEP. This discrepancy in TEP between different seawater living teleosts is most probably the result of differing ion selectivity’s of the TJs. Leaky epithelia, like in the fish proximal intestine, is in general more permeable to Na^+^ than to Cl^−^ which allows some of the Na^+^ transported by the NKA to the LIS to leak back to the intestinal lumen (Field et al., 1978; Loretz, 1995). This gives rise to a less positive (or negative) TEP depending on the balance between electroneutral (eg. NKCC2/NCC) and electrogenic transport (eg. Na^+^-driven nutrient uptake) across the epithelium (Jiang et al., 1998; Usami et al., 2001, 2003). Thus, the decrease in TEP seen in the proximal intestine with decreasing omega-3 levels further supports an increased Na^+^-permeability across the TJ as a result of low dietary EPA and DHA levels. A decrease in TEP caused by intestinal barrier impairment will in many instances be compensated for by an increased activity of the lateral NKAs in order to try to maintain an optimal osmolality in the LIS. However, no support could be found in the current study for such a compensation in increased active transport, neither as an increase SCC nor any effect mRNA levels of NKA1c.

Different fatty acids have direct impact on the function of the tight junction complexes and thus intestinal barrier function (Jiang et al., 1998; Usami et al., 2001, 2003). In the current study, increased levels of OA (oleic acid) and linoleic acid (LA) may provide a plausible explanation for the intestinal barrier impairment seen with this diet, as these FAs all have been connected to barrier dysfunction in mammalian model systems. Mammalian *in vitro* models have investigated the impact of specific FA on intestinal barrier function. LA, γ-linoleic acid (GLA) and ALA, but not OA, resulted in decreased TER, *i.e.* impaired barrier function of Caco-2 cells (Usami et al., 2001, 2003). In a vascular endothelial cell line, LA and OA decreased TER while ARA and ALA had no effect (Jiang et al., 1998). In contrast, GLA, dihomo- γ-linoleic acid (DGLA), and ARA, increased TER, *i.e.* strengthened the barrier, in the T84 human intestinal cell line whereas LA, ALA and DGLA had no effect (Willemsen et al., 2008). The results from these mammalian model systems and the present study suggest that different FA exert different effects in different types of cells. Also, the marine, long omega-3 FAs, EPA and DHA, were suggested to induce barrier dysfunction in Caco-2 cells (Usami et al., 2001, 2003). However, these negative effects maybe the result of lipid peroxidation as DHA supplemented with antioxidants mitigated the dysfunction (Roig-Pérez et al., 2004). In agreement, both DHA and EPA tightens the epithelium when human intestinal epithelial cells (T84) were supplemented with vitamin D and E (Willemsen et al., 2008). These data highlight that both the type and the quality of the fatty acids are crucial for maintaining the beneficial effects of PUFAs on intestinal barrier functions. Taking these previous observations in mammalian tissues and cell-lines into account suggest that the observed impairment of the intestinal barrier function in the current study may be due to the decreased relative levels of EPA and DHA in combination with increased relative levels of OA, ALA and ARA, with decreasing dietary levels of EPA and DHA in the feed.

Increased intestinal permeability is directly linked to intestinal inflammation in fish (Knudsen et al., 2008; Sundh et al., 2009, 2010, 2019; Niklasson et al., 2011; Venkatakrishnan et al., 2019) as in mammals, where impaired intestinal barrier function is one of several risk factors behind chronic intestinal inflammatory diseases (Michielan and D’Incà, 2015). Different fatty acids may also have indirect impact on the function of the tight junction complexes and thus intestinal barrier function through generation of secondary active compounds (Huang et al., 2020). The link between barrier dysfunction and relative level of different FAs such as ARA, is suggested to be through their effect on the inflammatory state. ARA is used as a substrate by cyclooxygenase (COX), lipoxygenase (LOX) and cytochrome P450 enzymes and converted into eicosanoids (Ricciotti and Fitzgerald, 2011). Increased levels of ARA in the cell membranes result in increased production of eicosanoid family molecules that can mediate pro-inflammatory processes, resulting in increased inflammation, a state that can be connected to intestinal barrier dysfunction in mammals including humans (Ricciotti and Fitzgerald, 2011; Huang et al., 2020). In the current study, ARA doubled in the intestinal FAs in the low compared to high omega-3 levels. This suggests that the reduced intestinal barrier function seen in the Atlantic salmon, at least in part, is a result of an increase in the pro-inflammatory FA, ARA. Yet another support for the correlation between long chain omega-3 FA and reduced inflammation in mammals is that increased levels of DHA and EPA decreases ARA in mammals (Calder, 2017). This may reduce a pro-inflammatory reaction through decreased production of potent eicosanoids, and possibly also through reduced production of pro-inflammatory cytokines, also known to induce intestinal barrier dysfunction (Calder, 2017). Further studies are however needed to elucidate if the observed intestinal barrier dysfunction of Atlantic salmon fed low, 0.5%, omega-3 levels also is mediated by increased pro-inflammatory reactions as a result of increased eicosanoid production from ARA.

The molecular mechanism behind epithelial permeability and selectivity across the intestine is determined by the main intercellular tight junction proteins, claudin, occludin and tricellulin (Chasiotis et al., 2012; Sundell and Sundh, 2012; Kolosov et al., 2013; Sundh and Sundell, 2015). Thus, another possible explanation for the barrier effects of dietary omega-3 levels is through changed expression of specific tight junction proteins. Mammalian claudins are broadly divided into barrier- or pore-forming isoforms, where the specific character is attributed to their effect on specific anions or cations (Krug et al., 2014; Krug, 2017; Rosenthal et al., 2017). To date, there are very few functional studies regarding the selectivity and permeability characteristics of fish claudins in general and none regarding Atlantic salmon intestinal claudins. However, Tipsmark et al. (2010a) suggested that claudin-25b is a barrier forming isoform and claudin-15 a pore forming isoform in the Atlantic salmon intestine. These suggestions are based on a sequence analysis comparing the Atlantic salmon claudins to mammalian and zebrafish claudins, which both were verified to be ion selective. Based on this assumption, the increased TER in both intestinal regions after seawater transfer may be due to an up-regulation of a barrier-forming claudin, which is also supported by the drastic up-regulation of claudin-25b in seawater. On the other hand, claudin-15, a suggested pore-forming isoform, also increased in response to the seawater acclimation in the proximal intestine concomitant with increased TER, which makes this assumption elusive. In addition, no dietary effects were observed in the expression of tight junction proteins, therefore the claudin-25b or claudin-15 differential expression patterns cannot explain the impaired barrier function seen in response to low EPA and DHA levels in the feed.

Occludin and tricellulin are both important tight junction proteins contributing to the barrier against uncharged macromolecules and mediating an increase of the TER (Krug, 2017). This is supported by studies using the mammalian MDCK cell-line where overexpression of occludin resulted in increased TER and thus a tighter epithelium (McCarthy et al., 1996). The tendency towards decreased expression of occludin in the proximal intestine with low omega-3 levels, may suggest that occludin is involved in the barrier formation towards ions in this intestinal region. Interestingly, in the distal intestine of the Atlantic salmon there was an upregulation of the occludin expression in the lower, 1 and 0.5%, omega-3 groups concomitant with a lack of dietary effects on barrier function in this region of the seawater acclimated fish. This may suggest that the distal intestine is able to compensate for a reduction in TER caused by low the omega-3 levels through up-regulation of occludin.

In conclusion, this study shows that low levels of omega-3 in the diet impairs the intestinal functions and reduce Atlantic salmon health and welfare. The low inclusion level do not affect osmoregulation assessed as plasma ion levels, but it reduce growth and changes the FA composition of the proximal intestine. Low omega-3 dietary levels further results in decreased omega-3 content of the intestinal epithelial membranes as well as in impaired intestinal barrier function and decreased TEP in the proximal intestine. The potentially pro-inflammatory FA, ARA, on the other hand, increased with decreasing omega-3 dietary levels. Transfer of Atlantic salmon from freshwater to seawater in general increased the transcript levels of tight junction proteins concomitant with increased barrier function, assessed as TER.

## Data Availability

The original contributions presented in the study are included in the article/Supplementary Materials, further inquiries can be directed to the corresponding author.
